# Neighborhood-based analysis of adolescent hidradenitis suppurativa prevalence in a large metropolitan area

**DOI:** 10.1016/j.jdin.2022.08.015

**Published:** 2022-08-30

**Authors:** Carrie L. Vuong, Helen H. Park, Sijia Zhang, Rosalynn R.Z. Conic, George K. Hightower

**Affiliations:** a Department of Dermatology, University of California, San Diego, San Diego, California; b Division of Pediatric and Adolescent Dermatology, University of California, San Diego and Rady Children's Hospital, San Diego, California; c School of Medicine, University of California, San Diego, La Jolla, California; d Department of Family Medicine and Public Health, University of California, San Diego, La Jolla, California

**Keywords:** adolescence, health disparities, hidradenitis suppurativa, neighborhoods, pediatrics

To the Editor:

Hidradenitis suppurativa (HS) is a chronic, dermatologic illness increasingly recognized in the pediatric population. For adolescents with HS, pain and scars can result in long-lasting psychosocial distress and functional impairment. Existing data on HS demonstrate that prevalence varies widely by race/ethnicity but there is no clear explanation as to why.^1^ In particular, studies of pediatric population with HS often fail to explore race as a social determinant of health. In contrast, use of neighborhood-level data in conjunction with patient demographics, such as race, allows for the consideration of environmental and socioeconomic factors that may otherwise function as unexplored confounders.

To estimate the prevalence of adolescent population with HS at the neighborhood level in San Diego County, we performed an institutional review board-approved retrospective chart review of patients aged 15 to 19 years diagnosed with HS, seen between February 10, 2011, and February 10, 2021, at Rady Children’s Hospital. Individuals with *International Classification of Diseases, Tenth Revision* code-based diagnosis of HS were identified and their current home addresses were converted to a US Census Bureau defined census tract. Census tracts were then mapped onto specific neighborhoods (collections of census tracts) as defined by the city of San Diego.^2^ In regards to race/ethnicity, patients seen at Rady Children’s Hospital appear to be representative of the pediatric population in San Diego.^3^ To ensure patient confidentiality and reliability of prevalence estimates, neighborhoods with a low absolute number of cases (<30) were then excluded from further analysis. Neighborhoods with adolescent HS prevalence higher than estimates previously published by Garg et al^1^ were then characterized using available data on race/ethnicity, poverty, and Climate Equity Index (CEI) Score.^1^ The CEI score, developed by the city of San Diego, quantifies neighborhood access to opportunity using pollution burden and walkability among a total of 35 indicators.^4^

A total of 569 patients with HS (aged 15-19 years) were identified in 34 neighborhoods. Of these neighborhoods, only 7 met the threshold of >30 cases. Among these 7 neighborhoods, HS prevalence (per 100,000) was 409 for Latinos/Hispanics and 709 for African American/Blacks (Table I). These 7 neighborhoods have an above average percentage of adolescents living in poverty (Table I) and residents identified as Black/African American (13%) and Latino/Hispanic (62%) (Fig 1). In contrast, Blacks/African Americans and Latinos/Hispanics make up, respectively, 4% and 54% of San Diegans aged 15 to 19 years. All 5 neighborhoods for which CEI scores are available contained ≥1 census tracts identified as having below-average access to opportunity.

**Table I:** Neighborhood poverty and prevalence of adolescents living with HS

Neighborhood	HS cases	Adolescent population (15-19 y)	HS prevalence per 100,000	Adolescents living in poverty (%)∗
1	48	11,332	424	29.9
2	39	10,538	370	25.2
3	39	14,510	269	34.1
4	38	7814	486	27.6
5	38	11,657	326	19.6
6	34	7443	457	27.9
7	32	5829	549	14.2

*HS,* Hidradenitis suppurativa.

∗ Percentage of adolescents aged 12 to 17 years living below 100% Federal Poverty Level, as reported by the San Diego County. The average percent for ages 12 to 17 years in San Diego County overall is 16.8%.

**Fig 1:**
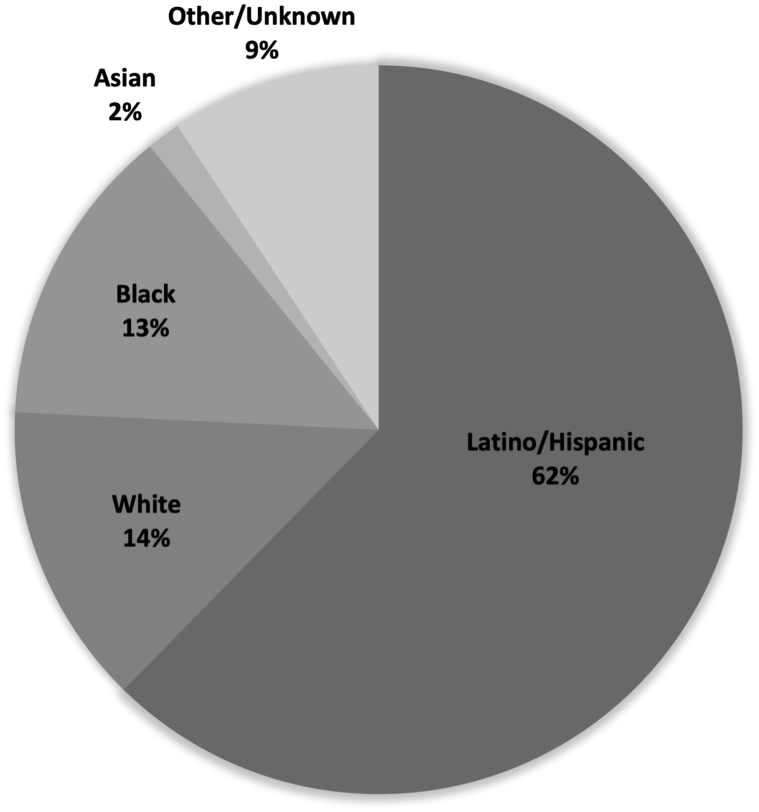
Race/Ethnicity in neighborhoods with a high prevalence of adolescents living with HS. For purposes of this study: (1) a patient is identified as Black/African American, if identified as such in the RCH electronic health record (EHR), (2) Latino/Hispanic if identified as such in the RCH EHR and did not also identify as Black/African American, (3) Asian if identified as such in the RCH EHR and not identified as Black/African American or Latino/Hispanic, and (4) White if identified as such in the RCH EHR and did not also identify as Black/African American, Latino/Hispanic, or Asian. *HS*, Hidradenitis suppurativa; *RCH*, Rady Children’s Hospital.

In the United States, racial residential segregation appears essential to understanding many observed racial disparities in health, yet to our knowledge, this study is the first to explore neighborhood-level data in adolescent patients with HS.^5^ Our findings raise important questions regarding how structural inequalities may impact the care and well-being of adolescents living with HS. HS prevalence is high among Black/African Americans; however, attributing this observation to genetic susceptibility may inappropriately bias future studies. Importantly, our aggregated data should not be applied to infer individual risk or correlation. Future studies are needed to examine early-life exposures and possible environmental factors that may impact the care and well-being of adolescents living with HS.

## Conflicts of interest

None disclosed.

## References

[1] Garg A., Wertenteil S., Baltz R., Strunk A., Finelt N. (2018). Prevalence estimates for hidradenitis suppurativa among children and adolescents in the United States: a gender- and age-adjusted population analysis. J Invest Dermatol.

[2] San Diego Region 2010 CENSUS TRACTS with Subregional Areas. San Diego Association of Governments. https://www.sandag.org/uploads/publicationid/publicationid_1738_15634.pdf.

[3] Park H.H., Conic R.R.Z., Zhang S. (2021). Oral glycopyrrolate for primary focal hyperhidrosis in a pediatric population: a cross-sectional study. JAAD Int.

[4] The City of San Diego’s 2015 Climate Action Plan. *2019 San Diego’s Climate Equity Index Report*. 2019. Accessed January 25, 2022. https://www.sandiego.gov/sites/default/files/2019_climate_equity_index_report.pdf

[5] Williams D.R., Collins C. (2001). Racial residential segregation: a fundamental cause of racial disparities in health. Public Health Rep.

